# Decellularized Extracellular Matrix as an *In Vitro* Model to Study the Comprehensive Roles of the ECM in Stem Cell Differentiation

**DOI:** 10.1155/2016/6397820

**Published:** 2015-12-06

**Authors:** Takashi Hoshiba, Guoping Chen, Chiho Endo, Hiroka Maruyama, Miyuki Wakui, Eri Nemoto, Naoki Kawazoe, Masaru Tanaka

**Affiliations:** ^1^Frontier Center for Organic Materials, Yamagata University, 4-3-16 Jonan, Yonezawa, Yamagata 992-8510, Japan; ^2^Tissue Regeneration Materials Unit, International Center for Materials Nanoarchitectonics, National Institute for Materials Science, 1-1 Namiki, Tsukuba, Ibaraki 305-0044, Japan; ^3^Graduate School of Science and Engineering, Yamagata University, 4-3-16 Jonan, Yonezawa, Yamagata 992-8510, Japan; ^4^Institute for Materials Chemistry and Engineering, Kyushu University, 744 Motooka, Nishi-ku, Fukuoka, Fukuoka 819-0395, Japan

## Abstract

Stem cells are a promising cell source for regenerative medicine. Stem cell differentiation must be regulated for applications in regenerative medicine. Stem cells are surrounded by extracellular matrix (ECM) *in vivo*. The ECM is composed of many types of proteins and glycosaminoglycans that assemble into a complex structure. The assembly of ECM molecules influences stem cell differentiation through orchestrated intracellular signaling activated by many ECM molecules. Therefore, it is important to understand the comprehensive role of the ECM in stem cell differentiation as well as the functions of the individual ECM molecules. Decellularized ECM is a useful *in vitro* model for studying the comprehensive roles of ECM because it retains a native-like structure and composition. Decellularized ECM can be obtained from *in vivo* tissue ECM or ECM fabricated by cells cultured *in vitro*. It is important to select the correct decellularized ECM because each type has different properties. In this review, tissue-derived and cell-derived decellularized ECMs are compared as *in vitro* ECM models to examine the comprehensive roles of the ECM in stem cell differentiation. We also summarize recent studies using decellularized ECM to determine the comprehensive roles of the ECM in stem cell differentiation.

## 1. Introduction

Stem cells are a promising source of cells for tissue engineering and regenerative medicine. Regenerative medicine applications that utilize stem cells require the regulation of stem cell differentiation [1, 2]. Stem cell differentiation is regulated by transcription factors that are expressed in specific tissues and organs [3–5]. During stem cell differentiation, the expression of transcription factors is regulated by signals from the extracellular microenvironment, including soluble factors and the extracellular matrix (ECM). Previous research determined that the ECM influences stem cell differentiation and the maintenance of stemness [6–9]. Therefore, it is important to clarify the role of the ECM in stem cell differentiation.

The ECM is a complex structure composed of collagens, proteoglycans, glycoproteins, and glycosaminoglycans. Researchers have studied the effects of individual ECM components on stem cell differentiation by coating single ECM proteins and observing the phenotypes of genetically mutated animals and cells. However, the ECM is composed of many types of proteins and glycosaminoglycans. Cellular functions, such as cell proliferation and differentiation, are precisely tuned by the combination of these molecules [10, 11]. Therefore, it is necessary to elucidate the comprehensive roles of the assembled ECM in stem cell differentiation as well as the roles of single ECM molecules. The* in vitro* ECM model is useful for these studies. However, it is difficult to construct* in vitro* ECM models using chemical and physical methods because of the complex composition of the ECM. Decellularized ECM is an alternative* in vitro* model that can elucidate the comprehensive roles of the ECM. In this review, we summarize the researches that have been conducted to understand the comprehensive roles of the ECM in stem cell differentiation using decellularized ECM as an* in vitro* ECM model.

## 2. General Roles of the ECM in the Regulation of Cellular Functions

**Figure 1:**
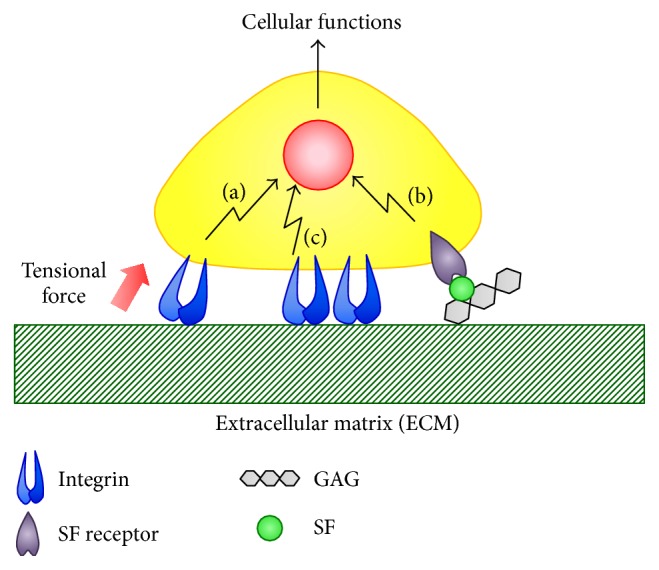
Three regulatory modes of cellular functions. (a) The mechanical stimulation from substrates of different stiffness. (b) Signal activation from soluble factors bound to ECM. (c) Signal activation from adhesion molecules such as integrins. SF indicates soluble factor and GAG indicates glycosaminoglycan.

The ECM is composed of many types of collagens, proteoglycans, glycoproteins, and glycosaminoglycans. These molecules are assembled to form a complex structure [10]. The protein components of the ECM vary for different tissues and organs [12]. The composition of the ECM is determined by developmental and pathological conditions [13–15]. The ECM acts as a substrate to facilitate cell adhesion for the formation of tissues and organs. The ECM is also a physical barrier between different tissues [16]. In addition to these physical roles (i.e., a substrate for cell adhesion and a physical barrier), the ECM influences many cellular functions through three modes: (a) mechanical stimulation from substrates with different stiffness, (b) regulation of soluble factor availability and activity, and (c) intracellular signaling activated by cell adhesion molecules (Figure 1).

The stiffness of tissues and organs varies according to their type. Stiffness is partially dependent on the ECM and can influence cellular functions [17–20]. Lo et al. reported that cells could recognize the stiffness of substrates. When cells were cultured on a gel substrate that contained both hard and soft regions, the cells migrated from the soft gel to the hard gel [17]. Mesenchymal stem cells (MSCs) can also recognize the stiffness of a substrate and differentiate into different lineages according to substrate stiffness [18].

ECM proteins can bind several soluble factors, such as bone morphogenetic proteins (BMPs) and Wnt protein, to regulate their activity. Wang et al. reported that BMP signaling was regulated by ECM molecules in* Drosophila*. Type IV collagen can bind the BMP signaling molecule Decapentaplegic (Dpp) to form a gradient pattern for correct* Drosophila* development [21]. Wnt protein can bind to ECM proteins such as proteoglycans [22]. The local concentration of Wnt protein increases by binding to the ECM. The accessibility to receptors is increased because of the increased availability of Wnt protein compared to soluble Wnt protein [23]. The ECM can downregulate the activity of soluble factors. Biglycan can bind to chordin, an inhibitor of BMP, to suppress BMP signaling [24].

Additionally, ECM proteins themselves can activate intracellular signaling through the interaction with cell adhesion molecules such as integrins [25]. Integrin activated intracellular signaling regulates various cellular functions such as cell survival, proliferation, morphogenesis, migration, and differentiation [25, 26]. Integrins are heterodimers composed of *α* and *β* chains. The combination of integrin heterodimers determines their ligand specificity [26]. Different combinations of integrin heterodimers activate different intracellular signaling pathways. For example, integrin *α*5*β*1 bound to fibronectin activates extracellular signal-regulated kinase (ERK), whereas integrin *α*3*β*1 bound to laminin-511/521 activates Akt rather than ERK [27]. Integrin signaling can cross talk with intracellular signaling activated by growth factors and can modulate their signaling [28]. Several interactions between cells and ECM and the signals activated by these interactions regulate cellular functions. Therefore, it is important to obtain a comprehensive understanding of how the ECM and individual ECM molecules influence stem cell differentiation.

## 3. Decellularized ECM as an* In Vitro* ECM Model

The composition of the ECM is complex and tissue-specific. It is difficult to obtain an* in vitro* ECM model that recreates the* in vivo* ECM composition by simple mixing of single ECM molecules. To solve this problem, decellularized ECM is used as an* in vitro* ECM model. Decellularized ECM is derived from the tissue and ECM proteins that are deposited by* in vitro* cultured cells [29–31]. Appropriate decellularization methods are required to obtain decellularized ECM that is similar (in composition and structure) to the original ECM. Decellularization is mainly performed with chemical, physical, and biological treatments and their combinations. Decellularized ECM is often cross-linked for stabilization. The effects of these treatments on the composition of the original ECM have been previously reviewed [29, 32]. The correct treatment should be selected for the preparation of decellularized ECM. The decellularized ECM source should also be considered for the preparation of decellularized ECM as an* in vitro* ECM model. We compared tissue-derived and cell-derived decellularized ECM.

### 3.1. Tissue-Derived Decellularized ECM

Tissues and organs are attractive sources of decellularized ECM because the composition of their decellularized ECM is identical to the composition of native ECM after correct decellularization treatments. Tissue-derived decellularized ECM is expected to exhibit native mechanical properties (e.g., stiffness) and microstructure. Tissue-derived decellularized ECM can be used in several forms, such as whole tissue/organ shapes [33, 34], patch-type shapes [35], coating material for two-dimensional (2D) cell culture substrates [36, 37], and injectable gel [38, 39]. Tissue-derived decellularized ECM is applied as an* in vitro* ECM model to regulate stem cell differentiation and to study the comprehensive roles of ECM in stem cell differentiation (Table 1).

Sliced brain sections can be decellularized, and this decellularized ECM can be used as the substrate for the three-dimensional (3D) culture of neural stem cells (NSCs) [40]. In decellularized brain ECM, NSCs can attach, proliferate, and retain their stemness [40]. Other researchers reported that decellularized brain ECM facilitated the differentiation of stem cells. Crapo et al. determined that the differentiation of NSCs and PC12 cells into neural cells was facilitated by exposure to decellularized brain ECM [41, 42]. Baiguera et al. reported that MSCs differentiated into neural cells on electrospun gelatin scaffolds that contained decellularized brain ECM [43]. Although the mechanism needs to be clarified, decellularized brain ECM is an attractive scaffold for nerve tissue regeneration.

Liver stem-like cells have been seeded on decellularized liver ECM to guide their differentiation into hepatocytes [44]. In decellularized liver ECM, liver stem-like cells lost the expressing embryonic marker genes encoding *α*-fetoprotein, nestin, nanog, and Oct3/4. The cells expressed hepatic genes encoding albumin and cytochrome P450s indicating their differentiation into hepatocytes. Moreover, liver stem-like cells can also form a subpopulation that expresses the genes encoding cytokeratin 19 and another subpopulation that expresses the genes encoding vimentin and CD31. This result suggests that liver stem-like cells can differentiate into epithelial-like and endothelial-like cells. Therefore, decellularized liver ECM can be used as an* in vitro* model of liver ECM for liver development.

Murine embryonic stem (ES) cells were seeded into the decellularized ECM derived from whole kidney [56]. In whole kidney-derived decellularized ECM, the ES cells lost their pluripotency and differentiated into a meso-endodermal lineage [56]. Murine ES cells were seeded into the decellularized ECM derived from whole lung [55]. In this decellularized ECM, the cells started to express thyroid transcription factor-1 (an immature lung epithelial cell marker) and prosurfactant protein C (a type II pneumocyte marker). These reports indicate that decellularized ECM can provide cues that direct stem cell differentiation toward specific lineages.

Although tissue-derived decellularized ECM has several advantages, several features of* in vitro* ECM models make them inferior for addressing the comprehensive roles of ECM in stem cell differentiation. One of the largest problems is the tissue source. It requires significant quantities of tissue-derived decellularized ECM to analyze ECM* in vitro*. It is difficult to obtain sufficient tissue-derived ECM from both animal and human sources.

In addition to the problem of tissue source, the isolation of small regions from tissue-derived decellularized ECM is difficult. Stem cells are maintained in small regions called niches in adult tissues* in vivo*. For example, NSCs are maintained in a basement membrane-like ECM called “fractones” that are adjacent to blood vessels in the subependymal layer of the lateral ventricle in the brain [70, 71]. Stem cell differentiation progresses in small regions in adult tissues [72, 73]. The composition of the ECM varies during the stem cell differentiation process, and the effects of the ECM on stem cell differentiation may change at each maturational stage [72, 73]. Therefore, the effects of the ECM on stem cell differentiation should be elucidated using an* in vitro* ECM model at each maturational stage. It is difficult to identify different maturational regions from tissue-derived decellularized ECM. It is difficult to analyze the comprehensive roles of ECM in stem cell differentiation at each maturational stage* in vitro* in tissue-derived decellularized ECM.

### 3.2. Cell-Derived Decellularized ECM

Cell-derived decellularized ECM is also used as an* in vitro* ECM model to regulate stem cell differentiation and to study the comprehensive roles of the ECM in stem cell differentiation (Table 2). Cells cultured* in vitro* can deposit ECM proteins beneath themselves [74, 75]. After the deposition of ECM proteins, the cells are specifically removed from the culture to obtain cell-derived decellularized ECM. Cell-derived decellularized ECM is an attractive* in vitro* ECM model. Abundant cell-derived decellularized ECM can be obtained for the* in vitro* analysis of the comprehensive roles of ECM in stem cell differentiation. It can be obtained in various forms, such as 3D structural scaffolds [63, 66] and 2D cell culture substrates [64, 65]. Cell-derived decellularized ECM can be obtained for use as an* in vitro* ECM model that is difficult to identify and isolate from tissue. Cell-derived decellularized ECM can model the stem cell niche and the ECM at each maturational stage.

The bone marrow stromal cell line MS-5 was cultured for the preparation of cell-derived decellularized ECM. The cell-derived decellularized ECM was used as the culture substrate for the* ex vivo* expansion of hematopoietic stem cells (HSCs) [69]. The MS-5 cells were cultured under different culture conditions (i.e., modulation of O_2_ tension and osteogenic induction). HSCs proliferated with expression of their specific surface markers on decellularized ECM obtained from cells cultured under low O_2_ tension. Proteomic analysis was performed to analyze the differences among four MS-5 cell-derived decellularized ECMs. The proteomic analysis revealed differential production of proteins such as aldehyde dehydrogenase and gelsolin. Cell-derived decellularized ECM can be obtained from cells cultured under specific conditions. Cell-derived decellularized ECM may reveal the conditions that form an ECM* in vivo*.

Decellularized ECM, derived from other cell types, was obtained for HSCs as an* in vitro* model for hematopoietic stem cell niche analysis [68]. Chan et al. prepared collagen particles encapsulating MSCs. Encapsulated MSCs were cultured under osteogenic conditions and then decellularized to obtain decellularized ECM. In this decellularized ECM, MSCs and HSCs were cocultured, and the suppression of HSC proliferation was observed. This result suggested that HSCs were maintained in a quiescent state. Intracellular signaling was examined to identify the mechanism that maintained HSC quiescence. The addition of a BMP2 neutralizing antibody increased the number of HSCs in the decellularized ECM, suggesting that BMP2 signaling is important for the maintenance of HSC quiescence. Cell-derived decellularized ECM may be a useful* in vitro* ECM model to elucidate the interactions in the HSC niche.

Although cell-derived decellularized ECM has several advantages over tissue-derived ECM in the* in vitro* analysis of the comprehensive roles of ECM in stem cell differentiation, it is difficult to obtain cell-derived decellularized ECM with the composition, mechanical properties, and microstructure which are identical to* in vivo* ECM. The composition of cell-derived ECM is dependent on the cell type and the cell culture conditions. The composition varies between decellularized ECM derived from primary versus passaged chondrocytes, leading to different cellular functions [76]. Alveolar type II epithelial cells form a basement membrane- (BM-) like structure with cocultured fibroblasts or Matrigel. However, alveolar type II epithelial cells are unable to form a BM-like structure without these coculture conditions [77, 78]. The initial cell culture substrate used for the preparation of decellularized ECM can influence the function of cells cultured on it [79]. The cell culture conditions used for decellularized ECM preparation should be carefully determined. A summary of the differences between tissue-derived decellularized ECM and cell-derived decellularized ECM is reported in Table 3.

## 4. Cellular Functions on Tissue Development-Mimicking Matrices

Stem cells differentiate into somatic cells step-by-step both* in vivo* and* in vitro* [3, 4]. The ECM is dynamically remodeled at each maturational stage according to this stepwise stem cell differentiation process [80, 81].* In vitro* ECM models that mimic the composition of the native ECM at each maturational stage are necessary to gain a comprehensive understanding of the roles of the ECM in stem cell differentiation. Stem cell differentiation occurs in limited regions in adult tissues, and it is difficult to isolate such limited regions for the preparation of tissue-derived decellularized ECM. Cell-derived decellularized ECM is an excellent model for ECM that is difficult to identify and isolate from tissues. Therefore, cell-derived decellularized ECM is useful for the preparation of* in vitro* models of ECM surrounding stem cells and differentiating cells at each maturational stage. The ECM surrounding mesenchymal stem cells (MSCs) and differentiating MSCs has been thoroughly studied using cell-derived decellularized ECM. We summarized the research involving cellular functions on cell-derived decellularized ECM mimicking* in vivo* ECM surrounding MSCs and differentiating cells. We also summarized the trials analyzing intracellular signaling on decellularized ECM.

### 4.1. Stemness Maintenance of MSCs on Undifferentiated MSC-Derived Decellularized ECM

The ECM is an important component of stem cell niches that maintains a stem cell's undifferentiated state and its stemness [6, 7]. MSCs gradually lose their stemness through* in vitro* passage culture. The loss of stemness prevents the large-scale application of MSCs in regenerative medicine [61]. Chen and his colleagues used undifferentiated MSC-derived decellularized ECM to solve this problem [61, 82]. MSCs maintained their ability to differentiate into other cell lineages after* in vitro* passage culture on undifferentiated MSC-derived decellularized ECM. This ability was lost in passage culture on conventional plastic cell culture substrates. MSCs subcultured on conventional plastic substrates decreased their ability to differentiate after five passages and lost it completely after six to seven passages. Cells subcultured on undifferentiated MSC-derived decellularized ECM maintained this ability after seven passages [61]. Spontaneous and induced differentiation of MSCs was suppressed on undifferentiated MSC-derived decellularized ECM [64, 82]. MSC proliferation is promoted on undifferentiated MSC-derived decellularized ECM [64].

Undifferentiated MSC-derived decellularized ECM has been used as an* in vitro* model of the ECM in the MSC niche to analyze intracellular signaling. BMPs play important roles in the differentiation of MSCs. BMP signaling activation is suppressed on decellularized ECM to prevent the spontaneous and induced differentiation of MSCs [64, 82]. Wnt signaling is activated on decellularized ECM to suppress osteogenesis [64, 83].

Intracellular levels of reactive oxygen species were also suppressed to maintain MSC characteristics on decellularized ECM [84]. Cellular senescence* in vitro* is also involved in stemness maintenance [85]. Telomerase activity was retained at higher levels on decellularized ECM than on conventional plastic substrate to inhibit stem cell replicative senescence during* in vitro* culture [84]. However, the mechanisms underlying such phenomena are still unclear. The molecular mechanisms by which decellularized ECM promotes telomerase activity and suppresses cell senescence must be identified.

### 4.2. Differentiation of MSCs on Tissue Development-Mimicking Matrices

**Figure 2:**
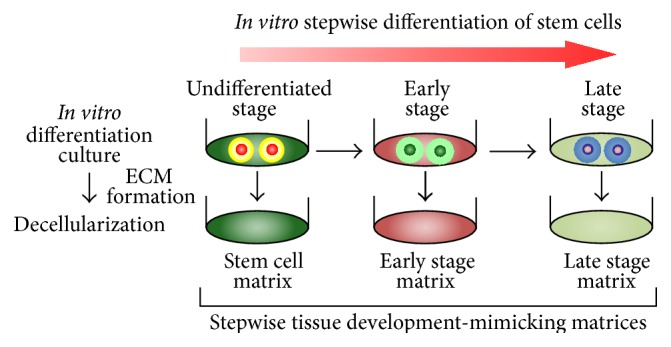
The concept and preparation procedure of stepwise tissue development-mimicking matrices.

MSC differentiation occurs step-by-step* in vitro* [86]. Therefore, it is possible that cell-deposited ECM can be obtained as decellularized ECM at each maturational stage. We have reported “stepwise osteogenesis-mimicking matrices” and “stepwise adipogenesis-mimicking matrices” as* in vitro* ECM models mimicking* in vivo* ECM at each stage of MSC osteogenesis and adipogenesis [64, 65, 83, 87]. These stepwise osteogenesis-mimicking matrices and stepwise adipogenesis-mimicking matrices were obtained through decellularization treatment of* in vitro* osteogenic and adipogenic MSC cultures. All decellularized ECMs were referred to as “stepwise tissue development-mimicking matrices” (Figure 2).

According to the progression of MSC differentiation, the composition of the ECM surrounding differentiating cells changes dynamically [88, 89]. This behavior suggests that ECM remodeling influences MSC differentiation. MSCs exhibit different osteogenic and adipogenic patterns on tissue development-mimicking matrices at different maturational stages. The osteogenesis of MSCs was only promoted on early osteogenic stage decellularized ECM. It was not promoted on decellularized ECM from late osteogenic or adipogenic stages [64, 87]. Conversely, the adipogenesis of MSCs was only promoted on early adipogenic stage decellularized ECM, and it was not promoted on decellularized ECM from late adipogenic or osteogenic stages [65, 87]. These results indicated that the differentiation of MSCs requires tissue- and stage-specific ECM [87].

**Figure 3:**
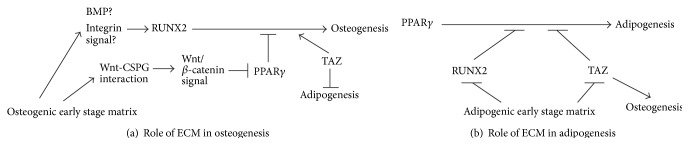
MSC differentiation mechanism on tissue development-mimicking matrices. (a) Role of tissue- and stage-specific ECM in the osteogenesis of MSCs. (b) Role of tissue- and stage-specific ECM in the adipogenesis of MSCs.

The molecular mechanisms of MSC differentiation on tissue development-mimicking matrices were examined [64, 65, 87]. MSC differentiation is controlled by transcription factors such as runt-related transcription factor 2 (RUNX2, also known as CBFA1), peroxisome proliferator-activated receptor *γ* (PPAR*γ*), and transcriptional activators such as transcriptional coactivator with PDZ-binding motif (TAZ) [5, 90, 91]. The expression levels of these molecules were measured on tissue development-mimicking matrices during the osteogenesis and adipogenesis of MSCs. The expression of* RUNX2*, which promotes osteogenesis, increased on decellularized ECM from early and late osteogenic stages but not on decellularized ECM from undifferentiated or adipogenic stages [64, 87]. The expression of* PPARG*, which promotes adipogenesis and inhibits osteogenesis, also increased on late osteogenic stage decellularized ECM [64, 87].* PPARG* expression was suppressed on decellularized ECM from undifferentiated and early osteogenic stages. These results suggested that early osteogenic stage ECM promotes the osteogenesis of MSCs and suppresses unexpected MSC differentiation (Figure 3(a)).

Similar to the osteogenesis of MSCs on tissue development-mimicking matrices, the expression patterns of transcription factors relating to adipogenesis differed for the different tissue development-mimicking matrices. Few differences in* PPARG* expression were observed during the adipogenesis of MSCs on tissue development-mimicking matrices. In contrast to* PPARG* expression,* RUNX2* expression levels increased on decellularized ECM from osteogenic stages but not that from adipogenic or undifferentiated stages [65, 87]. The expression level of* TAZ*, which promotes osteogenesis and inhibits adipogenesis, decreased only on decellularized ECM from the early adipogenic stage [65, 87]. Therefore, ECM at the early adipogenic stage demonstrated inhibitory effects on osteogenesis rather than stimulatory effects on adipogenesis (Figure 3(b)).

The regulatory mechanism of* PPARG* expression was examined on stepwise osteogenesis-mimicking matrices.* PPARG* expression was regulated by *β*-catenin signaling during the osteogenesis and adipogenesis of MSCs [92]. Intracellular *β*-catenin levels increased on decellularized ECM from undifferentiated and early osteogenic stages to suppress* PPARG* expression [64]. Intracellular *β*-catenin levels are regulated by canonical Wnt signaling [93]. However, the expression of Wnt signal-related genes such as* CTNNB1*,* LRP5*, and* WNT3A* did not change on decellularized ECM. Intracellular *β*-catenin levels decreased on decellularized ECM with treatment by chondroitinase ABC, which can remove chondroitin sulfate (CS) chains. Wnt protein can bind to CS chains to present to its receptors on cell surfaces [83]. The removal of CS chains decreased the availability of Wnt protein for the cells, and intracellular *β*-catenin levels decreased on the decellularized ECM treated with chondroitinase ABC.

Tissue development-mimicking matrices can be used as* in vitro* ECM models to study the comprehensive roles of ECM in stem cell differentiation and to clarify the intracellular signaling activated by interaction with the ECM.

### 4.3. Disadvantages to Cell-Derived Decellularized ECM 

Cell-derived decellularized ECM is a powerful* in vitro* model for analyzing the comprehensive roles of the ECM in stem cell differentiation. However, there are some disadvantages to using this substrate that require further investigation. The ECM is composed of many proteins and glycosaminoglycans produced by various cell types [12]. For example, the ECM in the NSC niches called “fractones” is adjacent to blood vessels, and endothelial cells and NSCs can supply ECM molecules for fractones [70, 71]. Therefore, the cell source for the preparation of cell-derived decellularized ECM should be considered carefully. The composition of the cell-derived decellularized ECM should be compared with the composition of the target ECM* in vivo*.

It is also important to understand how the effects of single ECM molecules on cellular functions are integrated to regulate stem cell differentiation. To answer this puzzling question, it is important to compare the effects between decellularized ECM and single ECM molecules on cellular functions.

## 5. Conclusions

Stem cells can exhibit different functions, such as proliferation and differentiation, on different types of decellularized ECM. Tissue-derived or cell-derived decellularized ECM can be used as an* in vitro* ECM model. The correct type of decellularized ECM should be selected because both decellularized ECM types have advantages and disadvantages. The results must be interpreted carefully from the viewpoint of similarity of decellularized ECM with the ECM* in vivo*. In spite of this consideration, decellularized ECM is one of the best ECM models mimicking native ECM composition and structure. Therefore, decellularized ECM is a powerful model for studying the comprehensive roles of ECM in stem cell differentiation.

## Figures and Tables

**Table 1:** Partial list of tissue/organ-derived decellularized ECMs for stem cell culture.

Target stem cell	Source of decellularized ECM	Application	Reference
Bone marrow-derived mesenchymal stem cells	Full thickness ear cartilage	Ear cartilage reconstruction	[45]
	Bladder	Bladder reconstruction	[46]
	Skin (dermal tissue)	Wound healing	[47]
	Brain	Neural differentiation	[43]

Adipose-derived mesenchymal stem cells	Adipose tissue	Adipose tissue engineering	[48]

Neural stem cells (NSCs)	Brain sliced section	NSC expansion with undifferentiated state	[40]
	Brain, spinal cord, urinary bladder	Neural differentiation	[41]

PC12 cell line	Brain, spinal cord, optic nerve	Neural differentiation	[42]

Adipose precursor cells	Placenta	Adipose precursor cell culture for adipose tissue engineering	[49]

Adipose stem cells	Tendon	Tenogenic differentiation	[50]

Endothelial progenitor cells	Umbilical cord artery	Vascular reconstruction	[51]

Liver stem-like cells	Liver	Hepatic differentiation, other epithelial-like and endothelial-like cells	[44]

Hair follicle stem cells	Skin (dermal tissue)	Hair bud-like structure formation and hair regeneration	[52]

Induced pluripotent stem (iPS) cells	Lung	Differentiation into lung progenitor cells	[53]
	Heart	Heart reconstruction	[54]

Embryonic stem (ES) cells	Lung	Lung reconstruction	[55]
	Kidney	Kidney reconstruction	[56]

**Table 2:** Partial list of cell-derived decellularized ECM for stem cell culture.

Target stem cell	Source of decellularized ECM	Application	References
Embryonic stem (ES) cells	Fibroblasts	Establishment of ES cells and maintenance of their stemness	[57]
	Differentiating embryoid body	ES cell proliferation and differentiation	[58, 59]
	HEK293	Pancreatic lineage differentiation.	[60]

Mesenchymal stem cells (MSCs)	Undifferentiated mesenchymal stem cells	Expansion culture with the maintenance of their stemness	[61]
	Osteoblasts	Osteogenic induction culture	[62, 63]
	MSCs under osteogenesis	Osteogenic induction culture and intracellular signal analysis	[64]
	MSCs under adipogenesis	Adipogenic induction culture and intracellular signal analysis	[65]
	Chondrocytes	Chondrogenesis	[66]

Hematopoietic stem cells (HSCs)	MSCs	Expansion culture with the maintenance of their stemness	[67]
	MSCs under osteogenesis	*In vitro* model of HSC niche	[68]
	MS-5 stromal cell line	*Ex vivo* expansion culture of HSCs	[69]

**Table 3:** Summary of the differences between tissue-derived and cell-derived decellularized ECM.

	Tissue-derived decellularized ECM	Cell-derived decellularized ECM
Advantages	Similar to native ECM composition, mechanical properties, and microstructure.	(i) Easy to obtain ECM model of small tissue regions. (ii) Possible for large-scale *in vitro *analysis.

Disadvantages	(i) Problems with ECM source. (ii) Difficult for large-scale *in vitro* analysis. (iii) Difficult to isolate small region from tissue.	Difficult to obtain decellularized ECM whose composition, mechanical properties, and microstructure are identical to native ECM.
